# Interoperability frameworks linking mHealth applications to electronic record systems

**DOI:** 10.1186/s12913-021-06473-6

**Published:** 2021-05-13

**Authors:** Kagiso Ndlovu, Maurice Mars, Richard E. Scott

**Affiliations:** 1 Department of Telehealth, School of Nursing & Public Health, College of Health Sciences, University of KwaZulu-Natal, Durban, South Africa; 2 Department of Computer Science, University of Botswana, Gaborone, Botswana; 3 College of Nursing and Health Sciences, Flinders University, Adelaide, South Australia Australia; 4 Department of Community Health Sciences, Cumming School of Medicine, University of Calgary, Calgary, Alberta Canada

**Keywords:** mHealth, eRecords, Interoperability Frameworks, Standards, eHealth Strategy, Botswana

## Abstract

**Background:**

mHealth presents innovative approaches to enhance primary healthcare delivery in developing countries like Botswana. The impact of mHealth solutions can be improved if they are interoperable with eRecord systems such as electronic health records, electronic medical records and patient health records. eHealth interoperability frameworks exist but their availability and utility for linking mHealth solutions to eRecords in developing world settings like Botswana is unknown. The recently adopted eHealth Strategy for Botswana recognises interoperability as an issue and mHealth as a potential solution for some healthcare needs, but does not address linking the two.

**Aim:**

This study reviewed published reviews of eHealth interoperability frameworks for linking mHealth solutions with eRecords, and assessed their relevance to informing interoperability efforts with respect to Botswana’s eHealth Strategy.

**Methods:**

A structured literature review and analysis of published reviews of eHealth interoperability frameworks was performed to determine if any are relevant to linking mHealth with eRecords. The Botswanan eHealth Strategy was reviewed.

**Results:**

Four articles presented and reviewed eHealth interoperability frameworks that support linking of mHealth interventions to eRecords and associated implementation strategies. While the frameworks were developed for specific circumstances and therefore were based upon varying assumptions and perspectives, they entailed aspects that are relevant and could be drawn upon when developing an mHealth interoperability framework for Botswana. Common emerging themes of infrastructure, interoperability standards, data security and usability were identified and discussed; all of which are important in the developing world context such as in Botswana. The Botswana eHealth Strategy recognises interoperability, mHealth, and eRecords as distinct issues, but not linking of mHealth solutions with eRecords.

**Conclusions:**

Delivery of healthcare is shifting from hospital-based to patient-centered primary healthcare and community-based settings, using mHealth interventions. The impact of mHealth solutions can be improved if data generated from them are converted into digital information ready for transmission and incorporation into eRecord systems. The Botswana eHealth Strategy stresses the need to have interoperable eRecords, but mHealth solutions must not be left out. Literature insight about mHealth interoperability with eRecords can inform implementation strategies for Botswana and elsewhere.

## Introduction

Adoption of eHealth (“use of information and communication technologies (ICT) for health”) [1] and mHealth (“the use of mobile communications for health information and services”) [2] is becoming commonplace globally. Changing expectations of patient populations, healthcare providers, and healthcare managers are moving countries towards continuous diagnosis and monitoring of health conditions irrespective of geographic location, making mHealth a promising alternative [3].

Developing countries with sufficient mobile network connectivity are increasingly adopting mobile technologies (e.g., phones, tablets, and applications [apps]) to complete tasks such as data collection, submission, and analysis as a way of strengthening their health systems [4, 5]. Over 40 developing countries use mHealth solutions such as the district health information system (DHIS2) tracker to support data management, reporting and mapping of surveillance data for HIV, TB and malaria programmes [6]. In Botswana mHealth interventions are facilitated by the high mobile phone penetration rate and improved ICT infrastructure [7]. The perceived impact of mHealth interventions include contributing to health system strengthening, ensuring equity, affordability, sustainability, discovery of new knowledge, and improvement of health outcomes and clinical decision making [7–9].

Similar to mHealth, the use and implementation of electronic records (eRecords) such as electronic health records (EHR), electronic medical records (EMR) or patient health records (PHR) is growing rapidly in developing countries. Indeed, the World Health Organization has identified data as the ‘fuel’ of eHealth, and that eRecords will become the basic building block of eHealth and a prerequisite for achieving Universal Health Coverage (UHC) [10]. However, major challenges with eRecords are fragmentation of data systems, duplicate functionality, large data sets in various locations, and non-uniform formats [11]. These impair the accurate reporting and decision making needed to address key healthcare challenges within both hospital and community-based delivery settings [8]. Although difficult to attain, interoperability of eRecord systems presents numerous benefits including improved patient management, quality of care, and decision making, and reduced healthcare costs [11].

Although mHealth and eHealth are promising options for primary healthcare [12, 13], the impact of such interventions can be greatly improved if the solutions are fully interoperable, allowing meaningful and seamless bi-directional transfer of data between these data sources. Interoperability deals with connecting systems and services through interfaces and protocols, using appropriate software engineering techniques and methods, to ensure efficient transfer and effective use of data [14]. Interoperability further involves many other aspects that must be considered: legislation, agreements between exchanging parties, governance, shared workflows, standardised data elements, semantic and syntactic choices, applications, technical infrastructure, safety, and privacy issues [11]. Such interoperability can be achieved at various ‘levels’ (technical, syntactic, semantic, organisational and legal) [14–16]. Guiding the process are interoperability frameworks that provide an agreed approach to achieve interoperability between organisations that wish to work together towards the joint delivery of services. The need for interoperability through the adoption of standards, interoperability architecture and an interoperability framework has been identified in the recently adopted Botswana’s National eHealth Strategy [17].

The aim of this study was to perform a literature review of published reviews of eHealth interoperability frameworks for linking mHealth solutions with eRecords, and to consider implementation approaches of these reports with respect to the needs expressed in Botswana’s National eHealth Strategy. Perspectives gained may also be of benefit to other developing nations.

## Methods

Literature searches were conducted using five databases: PubMed, EBSCOhost (specifically: ERIC, Academic Search Complete, e-Journals, Applied Science and Technology Index, Computers and Applied Sciences Complete, Medline), Web of Science, IEEEXplore, and Google Scholar. Broad key search terms were selected that encompassed the essential principles (eHealth, interoperability, standards, and data). Searches were restricted to: keywords only linked by the Boolean operator ‘AND’. For PubMed (“eHealth” AND “Interoperability” AND “Standards” AND “Data”), the period 1990-01-01 to 2020-03-31, and for reviews only (filter option for PubMed and Web of Science databases, manually during review for other databases). Only the first 100 results from Google Scholar were reviewed. Duplicates were removed, and titles and abstracts of each unique result reviewed by two authors (KN, RES) using the inclusion criteria: English language, review article, addressed one or more eHealth interoperability frameworks, and specifically addressed linking of mHealth devices to eRecords (e.g., EHR, EMR, PHR). Any disagreements were resolved by consensus. Full papers of selected resources were retrieved and reviewed by the same two authors against the same inclusion criteria, with disagreements resolved by consensus.

Included papers were reviewed to identify approaches for linking mHealth applications with an eRecord. Deductive coding was performed according to the process outlined by Linneberg and Korsgaard [18]. The four papers were read to identify issues considered to be important in the existing literature. A coding frame, a pre-defined list of descriptive codes, was developed by the first author and discussed by all authors, resulting in 21 codes (mHealth, mobile device, internet connectivity, EDGE, GPS, 2/3/4G, wireless sensors, user interface, Bluetooth, data, cloud services, radio-frequency, security, privacy, confidentiality, EHR, EMR, PHR, Interoperability Standards, Interoperability Framework, eHealth). Thereafter, the papers were systematically and iteratively searched through two cycles for elements able to inform mHealth and eRecord interoperability efforts in Botswana, and aligning these with the coding frame. These codes were then reviewed to refine the specifics of each and, through combination, reduced to a smaller number of higher-level themes, before analysing the available data and arranging them into four distinct themes (Infrastructure, Interoperability Standards, Data Security and Usability). These themes were then aligned to the interoperability levels addressed by each of the frameworks and findings summarised as narrative reflections on the current and future options for the interoperable information exchange between mHealth solutions and eRecord systems.

The Bostwana National eHealth Strategy document accepted on 10 March 2020 was reviewed to identify considerations relevant to mHealth and eRecords, and interoperability related to the two. The aspects of Botswana’s National eHealth Strategy related to interoperability were summarised and charted to align aspects of the literature review with the proposed development of an interoperability platform for the country. This included assessing the papers in line with the interoperability pillar of Botswana’s National eHealth Strategy.

## Results

**Fig. 1:**
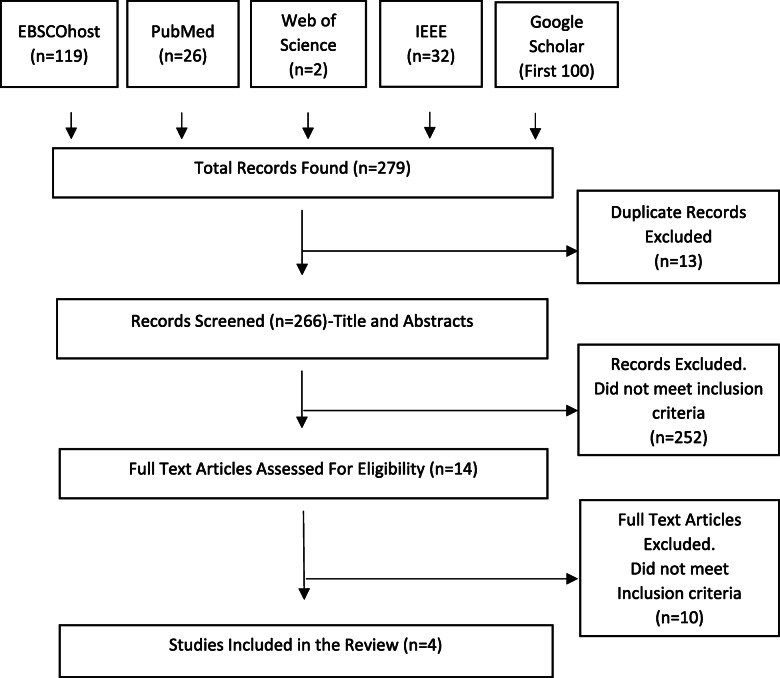
PRISMA flowchart for literature search

Of the 279 initial resources, four papers [19–22] met the inclusion criteria after removal of duplicates, screening, and review of full text papers, and were the subject of the review (Fig. 1). Each paper addressed an eHealth interoperability framework or a family of frameworks. These frameworks were the hierarchical XML-based Telemedicine Interoperability Framework Model (TIFM) [19], the X73PHD-IHE framework [20], the mobile health (MH) clinical decision support system (CDSS) framework [21], and the Ambient Assisted Living (AAL) frameworks [22]. Data extracted from the selected papers are summarised in Table 1.

**Table 1:** Purpose and description of review papers

Authors	Purpose of review	Approach	Interoperability Architecture/Platform
El-Sappagh et al. (2019), [21]. South Korea	A review of a cloud based comprehensive mHealth framework to support remote monitoring and management of type 1 Diabetes Mellitus.	Designed a distributed, semantically intelligent, cloud-based, and interoperable mHealth CDSS framework customizable to patient’s history and current vital signs. The proposed CDSS is based on the HL7 FASTO, a comprehensive OWL2 ontology, BFO, and clinical practice guidelines.	A comprehensive cloud-based architecture allowing interoperability across different service providers and different sources of medical data. The solution architecture provides four loosely coupled modules *(patient module, services module, cloud-based CDSS module, and the backend EHR systems module)*, but integrated based on ontology and the HL7 FHIR standard. Each module provides a particular set of functionalities. Therefore, changes in one module do not alter the architecture.
Adamko A, et al. (2016), [19]. Hungary	A review of a hierarchical XML-based TIFM aligned with international data exchange standards such as SNOMED and HL7.	Proposed a general accreditation scheme in accordance with SNOMED-CT and HL7 for personal Telemedicine Appliances coupled with an internationally standardised character code-table enabling international Telemedicine systems interoperability and a health data quality assurance measure.	A cloud based telemedicine architecture offering PaaS supporting IoT in a legal environment to the covered entities. The PaaS offers a full hardware architecture and software frameworks, allowing for quick access to needed resources.
Rubio ÓJ, et al. (2016), [20]. Spain	Review of the X73PHD-IHE based framework supporting a comprehensive IHE-based extension consisting of appropriate IHE profiles tailored to the needs of each eHealth and mHealth applications.	Assessed the risks of the X73PHD architecture, and proposed a cost-effective structure to provide support to the X73PHD domains to cope with the security and integration needs of different ehealth and mhealth applications. Further adopted appropriate IHE profiles to implement each layer, its translation into detailed modifications of the X73PHD models or framework and optimal algorithms to implement the cryptographic functions that would enhance the security of X73PHD.	A conceptual extended IHE-based X73PHD compliant healthcare architecture consisting of additive layers adapted to different eHealth and mHealth applications. The proposed features for each layer and the procedures to support them were carefully selected to minimize the impact on X73PHD standards on its architecture (in terms of delays and overheads).
Memon M et al. (2014) [22]. Denmark	Review to provide (1) an overview of the AAL concepts, (2) a survey of the current state-of-the-art in AAL frameworks, architectures, technologies and standards, and (3) an overview of current usage and real world deployment of specific AAL systems and platforms.	Conducted a literature survey of state-of-the-art AAL frameworks, systems and platforms to identify the essential aspects of AAL systems and investigate the critical issues from the design, technology, quality-of-service, and user experience perspectives. Also conducted an email-based survey for collecting usage data and current status of contemporary AAL systems.	i) SOA ii) A conceptual architecture consisting of four architectural layers, i.e. base, data, information, and context layers used for evaluation of the quality attributes of sensors, ambient data, and communication interfaces. iii) S3OiA offering a parallel view of architecture for connecting IoT devices for smart home applications and AAL systems using triple-space computing and RESTful web services. iv) The open service architecture which detects patients location using GIS services. v) ISO/EN 13,606 based standard architecture to transfer information among distributed medical systems. vi) Advanced cloud technology-based architecture which uses a DACAR platform, to enable controlled access to the clinical services for health monitoring.
*Abbreviations / acronyms*: *AAL* Ambient Assisted Living, *BFO* Basic Formal Ontology, *DACAR* Data Capture and Auto Identification Reference, *FASTO* Fast healthcare interoperability resources Semantic sensor network based Type-1 diabetes Ontology, *FHIR* Fast Healthcare Interoperability Resources,*GIS* Global Information System, *HL7* Health Level 7, *IHE* Intergrating the Healthcare Enterprise, *IoT* Internet of Things, *ISO/EN 13606* International Standards Organisation/Electronic Health Record Communication 13606, *OWL2* Web Ontology Language 2, *PaaS* Platform as a Service, *RESTful* Representational state transfer, *SNOMED-CT* Systematised Nomenclature of Medicine - Clinical Terms, *SOA* Service Oriented Architecture, *S3OiA* Three-layered Service Oriented Architecture, *TIFM* Telemedicine Interoperability Framework Model, *X73PHD-IHE* X73 Personal Health Device - Integrating the Healthcare Exchange, *XML* Extensible Markup Language			

The four papers also presented interoperability framework implementation approaches, which were charted under four common emergent themes: Infrastructure, Interoperability Standards, Data Security, and Usability. For each framework, the interoperability levels addressed and the corresponding thematic considerations were summarised (Table 2).

**Table 2:** Framework, interoperability level, and thematic considerations for the review papers

	Adamko et al., [19]	Rubio et al., [20]	El-Sappagh et al., [21]	Memon et al., [22]
**Framework**	XML-based TIFM	X73PHD-IHE Framework	Mobile health CDSS Framework	AAL Frameworks
**Interoperability Levels**	Syntactic, Semantic	Syntactic, Semantic	Syntactic, Semantic	Syntactic, Semantic
**Infrastructure Considerations**	Cloud services, private, public, hybrid and community, using SaaS, PaaS and IaaS	Cable and wireless setup of PHD “agents” (independent living devices) and aggregator devices called “managers” (smartphones, personal computers, personal health appliances, smart TVs etc.).	Comprehensive cloud based infrastructure supporting patient module, cloud-based CDSS module, backend EHR systems module, and mobile health services module	Interconnected medical sensors, WSANs, computer hardware, wired computer networks, software applications and databases.
**Interoperability Standards Considerations**	HL7	ISO/IEEE 11073 X73PHD	HL7 FHIR FASTO Ontology	HL7 ISO/IEEE 11073 ZigBee Bluetooth RFID IEEE 802.15.4
**Data Security Considerations**	HIPPA HITECH	Physical tokens for user authentication Additional password for user identification in the agent device Device certificate, signed by manufacturer Authentication by manager device Fingerprints in measurements Symemtric and Asymmetric encryption algorithms Frames encryption Secure transport layer Agent–manager authentication Role-based access control: Single-use encryption keys	N/A	RBAC and service based authorization Security and privacy policies for integrating homecare Apps with hospital systems using a TG Data encryption algorithms including DES and AES Semantic based access control for distributed identifiers, cross domain identity federation, multi-device credential management and context-aware access control.
**Usability Considerations**	Quick access to cloud resources pooled across multiple customers Metered services, allowing users easy tracking of platform usage and actual cost.	mHealth device self administration and sharing across users Automated real-time features Offline functionalities	Real-time feedback Decision support capabilities	Automatic connectivity feature Automatic seamless system updates Limited user interface screens Less error promts Auto-configurations for ready-to-use applications and devices User interface based on adaptive interactions
*Abbreviations / acronyms: AES* Advanced Encryption Standard, *DES* Data Encryption Standard, *HIPAA* Health Insurance Portability and Accountability Act, *HITECH* Health Information Technology for Economic and Clinical Health, *IaaS* Infrastructure as a Service, *ISO/IEEE 11073* International Standards Organisation/Institute of Electrical and Electronics Engineers 11073, *RBAC* Role Based Access Control, *RFID* Radio-frequency Identification, *SaaS* Software as a Service, *TG* Translation Gateway, *WSAN* Wireless Sensor and Actuator Network, *ZigBee* Zonal Intercommunication Global standard				

Framework characteristics, advantages, disadvantages and applicable conditions, were also summarised (Table 3).

**Table 3:** Framework, characteristics, advantages, disadvantages and applicable conditions

Framework	Characteristics	Advantages	Disadvantages	Applicable conditions
Telemedicine Interoperability Framework Model (TIFM)	Cloud based (PaaS) Telemedicine platform for secure remote access to health information by participatory entities and patients.	Easy access to patients information anywhere and anytime from any types of device. Usage and cost tracking feature. Support for wired and wireless data transmission methods while ensuring optimum speed, latency and availability.	XML-schemes and structure require agreements between entities and to be adapted to systems prior to data interchange. Semantic challenges for diseases identification tags during data exchange. Dependency on the vendor’s infrastructure and software, increases data security risks.	Remote patient monitoring and management over distributed network environments.
X73PHD-IHE framework	Enhancement of the security and interoperability features of the X73- PHD standards PHDs. A comprehensive IHE-based extension with layers adapted to supporting different eHealth and mHealth technologies.	Secure and robust, yet cost effective approach for PHDs, ideal for syntactic and semantic interoperability with other medical devices.	Limited specifications about IHE profiles required to implement interoperable eHealth/mHealth applications. Requires different levels of security and interoperability with each healthcare system.	Framework support is grouped in the domains of Health and Fitness, Independent Living and Disease Management.
Mobile health CDSS Framework	Realtime cloud based decision support mHealth solution utilising FASTO ontology to enhance knowledge quality and semantic interoperability with different EHR systems.	Remote collection, formalizing, integration, and analyzing of patient data through body sensors. Offers a complete, personalized, and medically intuitive care plans and sub-plans based on patient profiles.	The framework however lacks in addressing patient data security considerations.	The cloud-based solution is ideal for remote monitoring and management of medical conditions such as type 1 diabetes mellitus.
The Ambient Assisted Living (AAL) frameworks	An ecosystem of medical sensors, computers, wireless networks and software applications for remote healthcare monitoring in an Ambient Assisted environment.	Support for personalized, adaptive, and anticipatory features, necessitating high quality-of-service to achieve interoperability, usability, security, and accuracy.	Security, privacy, reliability, and robustness are perceived as main challenges. Requires more technical, economical, and multi-organizational resources and commitment to succeed. Usability is an issue since end-users who are mostly elderly, and disabled, have no technical expertise in handling different devices, applications, network equipment, gateways, and other infrastructural components.	Application of ICT technologies in personal healthcare and telehealth systems for countering the effects of growing elderly population. The primary goal being to extend the time which elderly people can live independently in their preferred environment using ICT technologies for personal healthcare.

## Review of the Botswana eHealth strategy

The Botswana e-Health strategy [17] was developed over several years and informed by broad literature and consultations. The final version was formally released March, 2020. The Botswana eHealth strategy [17] aligns with the Seventy-First World Health Assembly (WHA) Resolution (WHA71.7) on Digital Health adopted by WHO Member States in May 2018. [23]. It further aligns with key national policies including the Data Protection Act and the Data Management Policy. Absent from the eHealth Strategy is consideration of linking mHealth interventions with eRecord systems. None of the terms ‘mHealth’, ‘personal health record’, eRecord nor e-Record are used within the document. Although briefly addressed, the eHealth Strategy recognises the potential of emerging technologies utilising mobile devices, IoT, machine learning, artificial intelligence (AI), television white-spaces (TVWS) and sensors to populate health information systems with data. Moreover, capacity building and usability of all systems (user friendly interfaces, availability, performance capabilities) are emphasised. ‘Electronic health record’ is identified only in a list of acronyms but not in the main text. EMR is identified in the list of acronyms and once within the main text, with the abbreviation ‘EMR’ appearing three times in the main text or Tables. It is stated that *“All public hospitals have an Electronic Medical Record (EMR) that is real time, with much higher coverage”.* EHR is mentioned seven times, once identifying “*EHR and patient summary records*” as “*priority projects*”, and several times in relation to “*Establish*[ing] *a home-grown EHR for Botswana*” as a strategic intervention within a National eHealth Platform to be established by 2023. One activity contributing to this, and to be completed by 2021, is to “*Evaluate existing software solutions and establish a roadmap for transitioning them to the EHR roadmap*”. Hinting at interoperability issues, it is stated *“There is duplication of efforts (EMR and DHIS2 data), data coming from the same source and some of the software are not in real time.”* The importance, need, and value of interoperability is made clear in the strategy, with ‘Standards and Interoperability’ being identified as: (a) one of seven priority areas for development (page 8), (b) a strategic pillar (pages 29, 36), with “*the need for more interoperability and consolidation of existing information systems”* being noted (page 16), and the availability of “*interoperability architecture tools such as the Open Health Information Exchange (OpenHIE) and the Open Health Information Mediator (OpenHIM)*” (page 18) being recognised; and (c) description as a strategic objective, and establishment of an interoperability architecture / framework using the OpenHIM layer (subsection 3.5.4 Standards and Interoperability, pages 23/24).

## Discussion

The significant risks of having eRecords that are not interoperable was noted in Europe more than a decade go: *“Without the meaningful sharing and exchange of information, the gains would be marginal and not justify the cost of investments”*[24]. This statement remains valid and is even more pertinent in the developing world with limited financial resources for health. Botswana’s eHealth Strategy identifies the need for a homegrown EMR, and recognises the use of telemedicine and mHealth [17]. The Strategy also identifies the need for an interoperability platform and common HIS standards. However, the Strategy does not address interoperability between mHealth devices and eRecords, despite the anticipated expanded use of mHealth [23]. Although limited in their scope and application, four frameworks were found from the literature review that addressed linking of mHealth to eRecords [19–22]. Each of these frameworks is limited in satisfying the current interoperability needs of Botswana. Nonetheless to guide Botswana, and other developing countries with a similar dilemma, several lessons and options have been distilled from the literature to help avoid any inadvertent and unnecessary re-invention.

Although four themes were derived from the literature findings, interoperability is frequently described in terms of five ‘levels’: technical, syntactic, semantic, organisational, and legal [14–16]. From the literature review four themes were identified: infrastructure, interoperability standards, data security, and usability. For clarity, the themes and levels were mapped to one another in the following manner. ‘Infrastructure’ and ‘security’ mapped to all five levels of interoperability, ‘standards’ mapped to all except technical interoperability, and ‘usability’ mapped to only organisational interoperability.

Different infrastructure supported linking of mHealth to eRecords. Cloud-based infrastructure presented several benefits including flexibility to choose from private, public, or hybrid services tailored to specific user needs [19, 21]. These cloud services used SaaS, PaaS and IaaS, and were efficient for multiple user access and easy acquisition of resources from multiple stakeholders [19]. PaaS also allowed flexible access to health services via desktop, laptop or mobile devices, enabling anywhere and anytime access to data [19]. Cloud based mHealth infrastructure supported integration of CDSS capability for remote monitoring and management of patients with chronic diseases [21]. The efficiency of the CDSS solution was enhanced by using FASTO ontology [21]. Communication between eRecords developed on diverse platforms was addressed through the Common Object Request Broker Architecture (CORBA) and the Distributed Component Object Model (DCOM) [19]. Of interest, none of these papers discussed legal concerns such as the storage of patient sensitive information on servers in other countries, or access and ownership of data in non-State owned servers, associated with cloud-based services [19, 21].

Various interoperability standards supported linking mHealth with eRecords, in particular the Health Level 7 Fast Healthcare Interoperability Resources (HL7 FIHR) standard, and the ISO/IEEE 11073 Personal Health Device (X73*-*PHD) standard (as part of ISO/IEEE 11073 family of standards) described below [19–22]. HL7 FHIR offers definitions of how EHR data should be structured, semantically described, and communicated, and it works well with existing medical terminologies (SNOMED CT (SCT), LOINC, ICD, RxNorm, and UMLS). Moreover, the HL7 FHIR standard is based on HTTP and RESTful services, combining the best characteristics of HL7’s v2, v3, and clinical document architecture (CDA) [21]. HL7 FHIR defines 116 generic types (i.e. form templates) of interconnecting resources for all types of clinical information. It also defines four paradigms for interfacing between systems, including RESTful API, documents, messages, and services [21].

The ISO/IEEE 11073 X73-PHD standard brings the flexibility to define Integrating the Healthcare Enterprise (IHE) profiles with enhanced security features for servicing different mHealth devices and healthcare scenarios [20]. The X73-PHD component of the standards (11073 Personal Health Device) promotes the need for an openly defined, independent standard for controlling information exchange to and from personal health devices and other medical devices (e.g., cell phones, personal computers and personal health appliances). As an example, an end-to-end standard-based patient monitoring solution was utilised to transform medical data from the X73 Point of Care Medical Device Communication (PoC-MDC) devices into the EN13606 standard and stored the data at an EHR server [22].

Timely, accurate and secure patient information is fundamental to meeting key health indicators such as the Sustainable Development Goals (SDGs) [25]. To address timely data transfers, El-Sappagh et al., recommended JSON RESTful messages given their capability to support relatively small data sizes [21]. Rubio et al., enhanced security of their ISO/IEEE 11703 X73PHD standard through custom IHE profiles with layers (layer 0.x – 2.x) addressing different security concerns [20]. They further categorised security according to user, agent and manager device risks [20]. mHealth security measures (authentication, authorisation mechanisms, encryption algorithms (e.g. DES and AES), and data transfer security (e.g. secured network transport layer) were recommended [20, 22].

Usability is an important component for mHealth solutions if they are to effectively serve the needs of the target users [20–22]. Indeed Rubio et al. stated users should be able to easily take their biomedical measurements with any peripheral device [20]. Similarly, El-Sappagh et al. reported that usability, real-time feedback, and decision support capabilities in telemedicine systems are crucial [21].

### Guidance

The authors used prior knowledge and experience to envision appropriate use cases that would demonstrate the need for linking mHealth applications with eRecord systems. Use cases of relevance to Botswana include (1) mHealth data collected within a hospital transferred to the hospital’s EMR; (2) remote patient monitoring data from mobile devices transferred to the patient’s eRecord(s); (3) cellphone-based teleconsultation supporting realtime transfer of images for specialist review; (4) surveillance data collected by community healthcare workers using mobile devices and transferred to a central repository. These examples highlight that various sources and types of information will need to be exchanged and that mHealth interoperability with eRecords must be resolved for Botswana’s eHealth Strategy to move forward efficiently and effectively. Based upon the literature findings, the following guidance is proffered.

Despite recognised issues with both approaches, a mix of cloud-based and on-site infrastructure for linking of mHealth to eRecords is a key recommendation. However, cloud services were reported to encounter computing and bandwidth capacity challenges, and their proprietary nature could leave decision-makers with minimal information regarding their security configuration, both negatively affecting their acceptance [19]. This applies to Botswana where uncertainty exists around cloud-hosted services and could be addressed through increased sensitisation about cloud-services. SOA, where services are used only when needed (Apple’s Healthkit [26]), would be suitable for Botswana. Also, Quality of Service techniques to manage data traffic and reduce packet loss, latency and jitter, are recommended for efficiency.

Interoperability standards recommended for Botswana include HL7 FHIR and the ISO/IEEE 11703 X73PHD standards, and these are already referenced in Botswana’s eHealth Strategy. HL7 FIHR is expected to provide an easier, cheaper, and faster route to achieving interoperability. Botswana should also leverage common open interoperability interface standards (OpenHIE and OpenHIM) which were similarly referred to in Botswana’s eHealth strategy [17]. As supported by the literature, Botswana plans to have an eHealth/mHealth standards accreditation body [19]. Similar fora exist that might be exemplars (e.g., the Health Information Technology Standards Panel, the National Library of Medicine, and Canada Health Infoway). Botswana should leverage these existing structures while investigating costs associated with standards adoption, priorities, and local capacity needed for continuous monitoring and evaluation. mHealth standards for use of wireless sensor networks including ZigBee, Bluetooth, RFID, IEEE 802.15.4 should be considered and regulated locally to effectively support remote health monitoring applications [22].

Insecure health data compromises the goals of truly ‘informed health care’, and appropriate security measures would instill confidence in mHealth and eRecords users [20]. Botswana has recently developed a Data Protection Act – Act No.32 of 2018 (“the DPA”). Assented to by Parliament on 3rd August 2018 the Act is currently on notice, awaiting commencement. Thereafter, the DPA will provide the necessary safeguards related to the right to privacy of individuals and the collection and processing of personal data in Botswana, including issues such as cross-border transfer of data.

Usability must be high for optimal functionality. Given the proposed Botswana home-grown EHR, it must be able to simply and easily link with mHealth solutions. A user-centric and participatory approach coupled with fast prototyping and interactive feedback is recommended. Any interoperability features must be straightforward to the user(s) who must actively particpate in their selection and, after minimal training, be able to use the personal health devices without technical support as highlighted by Memon et al. [22]. The authors also recommend that mHealth solutions provide automatic connectivity features, support seamless system updates, and offer limited user interface screens, less error prompts, and auto-syncing with eRecords.

Another consideration for Botswana is system user interfaces based on adaptive interactions to enhance usability over time. Cloud-based platforms could offer flexible services for Botswana allowing for usage and cost tracking [19]. These could be enhanced to support offline and automated syncing functionalities.

Although not found in the review articles, other eHealth Strategy documents and country level guidelines supporting linking of mHealth solutions to eRecord systems also exist, and these could add value to Botswana’s eHealth Strategy [27–30].

## Conclusions

mHealth initiatives are growing in number, particularly in the developing world. Their linkage to one or multiple eRecord systems (EHRs, EMRs, PHRs) is desirable, yet is seldom acknowledged or addressed in the literature. Poorly planned or absent interoperability could result in deployment issues, unsuccessful implementations, poor user interfaces and experiences, security threats, and raised investment costs. This study has identified insights from the academic literature that can be leveraged to inform country-level eHealth Strategies such as Botswana’s. Four eHealth frameworks have been identified that addressed interoperability of mHealth and eRecord systems and that can provide guidance to Botswana and other developing countries with a similar dilemma. Applying the lessons and options discussed will strengthen eHealth Strategies and avoid unnecessary re-invention. The Botswana eHealth Strategy speaks to establishing a standards and interoperability framework and interoperability architecture during 2020 with a view to publishing and implementing them in 2021. It is essential that in doing so consideration is given to interoperability of mHealth applications and eRecords. This study is timely, and the findings and recommendations will raise awareness of the issue, as well as help inform and guide the resolution process.

## Data Availability

Not applicable; all data publicly available.

## Abbreviations

AAL: Ambient Assisted Living
AES: Advanced Encryption Standard
AI: Artificial Intelligence
BFO: Basic Formal Ontology
CDSS: Clinical Decision Support System
CORBA: Common Object Request Broker Architecture
DACAR: Data Capture and Auto Identification Reference
DCOM: Distributed Component Object Model
DES: Data Encryption Standard
DHIS2: District Health Information System vs 2
eHealth: Electronic Health
EHR: Electronic Health Record
EMR: Electronic Medical Record
eRecords: Electronic Records
ERIC: Education Resources Information Center
FASTO: Fast healthcare interoperability resources Semantic sensor network based Type-1 diabetes Ontology
FHIR: Fast Healthcare Interoperability Resources
GIS: Global Information System
HL7: Health Level 7
HIPAA: Health Insurance Portability and Accountability Act
HITECH: Health Information Technology for Economic and Clinical Health
HTTP: Hypertext Transfer Protocol
ICD: International Classification of Disease
ICT: Information Communication Technology
IEEE: Institute of Electrical and Electronics Engineers
IEEE 802.15.4: Institute of Electrical and Electronics Engineers 802.15.4 Standard
IHE: Intergrating the Healthcare Enterprise
IaaS: Infrastructure as a Service
IoT: Internet of Things
ISO/IEEE 11073: International Standards Organisation/Institute of Electrical and Electronics Engineers 11073
ISO/EN 13606: International Standards Organisation/Electronic Health Record Communication 13606
LOINC: Logical Observation Identifiers Names and Codes
mHealth: Mobile Health
OpenHIE: Open Health Information Exchange
OpenHIM: Open Health Information Mediator
OWL2: Web Ontology Language 2
PaaS: Platform as a Service
PHR: Patient Health Record
RESTful: Representational state transfer
RBAC: Role Based Access Control
RFID: Radio-frequency Identification
RxNorm: Normalized Names and Codes
SaaS: Software as a Service
SDGs: Sustainable Development Goals
SNOMED SCT: Systematized Nomenclature of MedicineStandard Clinical Terminologies
SOA: Service Oriented Architecture
S3OiA: Three-layered Service Oriented Architecture
TG: Translation Gateway
TIFM: Telemedicine Interoperability Framework Model
TVWS: Television White Space
UHC: Universal Health Coverage
UMLS: Unified Medical Language System
WSAN: Wireless Sensor and Actuator Network
X73PHD-IHE: X73 Personal Health Device - Integrating the Healthcare Exchange
XML: Extensible Markup Language
ZigBee: Zonal Intercommunication Global standard
